# Advances in Nutrient-Rich Foods for a Healthy Diet

**DOI:** 10.3390/foods12152946

**Published:** 2023-08-03

**Authors:** Sandra González-Palacios, Juristo Fonollá

**Affiliations:** 1 Unidad de Epidemiología de la Nutrición, Departamento de Salud Pública, Historia de la Ciencia y Ginecología, Universidad Miguel Hernández (UMH), 03550 Alicante, Spain; 2 Instituto de Investigación Sanitaria y Biomédica de Alicante, Universidad Miguel Hernández (ISABIAL-UMH), 03010 Alicante, Spain; 3 CIBER Epidemiología y Salud Pública (CIBERESP), Instituto de Salud Carlos III, 28034 Madrid, Spain; 4 Departamento de Nutrición y Bromatología, Universidad de Granada (UGR), 18071 Granada, Spain; juristo@ugr.es

The nutrient-rich foods are foods with a high content of protein, fiber, vitamins and minerals, but also a low content of saturated fats, added sugar and sodium [1]. Some examples are vegetables, fruits, nuts or olive oil among others. Moreover, some nutrient-rich foods can also present other bioactive compounds that appear to have beneficial health effects such as polyphenols. Due to their nutritional and bioactive composition, nutrient-rich foods usually are considered part of healthy dietary patterns.

In the last decades, the number of studies that evaluated the effect of nutrient-rich foods on health is constantly growing. A large number of foods can be considered nutrient-rich foods and a wide variety of health outcomes (beneficial or harmful) can be related to them. That is the reason why this topic is wide and diverse. The Special Issue “Nutrient-rich foods for a healthy diet” has the objective to unify the novelty evidence on how the consumption of nutrient-rich foods, their food composition, the properties of nutrients or their bioactive compounds contribute to health and well-being.

This Special Issue is composed of seven different manuscripts, five original research articles and two reviews and they can be assigned to three sub-topic groups.

One portion of the studies investigated food transformation to improve food composition of natal plum juice [2] and lupin-fortified bread [3]. On the premise that co-encapsulate probiotics and bioactive compounds ensure timely delivery in the gastrointestinal tract and their health effects. Seke et al. [2] evaluated the co-encapsulation of Natal plum (*Carissa macrocarpa*) juice inoculated with *Lactiplantibacillus plantarum* 75 (*Ltp. plantarum* 75) by freeze-drying using pea protein isolate, maltodextrin, and psyllium mucilage. Their results showed that microencapsulation is important for improving stability and allowing for the development of functional foods. Regarding lupin-fortified bread, Plustea et al. [3] evaluated the nutritional, phytochemical, sensory, and rheological properties of wheat flour dough and bread under a replacement by lupin flour at 10, 20, and 30%. The results showed an improvement in the nutritional properties (proteins, lipids, and mineral) of bread with addition of lupin flour, especially in the bread with 30% lupin flour. However, in the sensory analysis, the most highly appreciated by consumers was the bread with 10% lupin flour. This study shows that a change in flour composition can increase the nutritional properties of bread with high consumer acceptance.

Another portion of the studies from this Special Issue is mainly focused on nutrient composition and their bioaccessibility in some foods. On one hand, Ciudad-Mulero et al. [4] evaluated the content and bioaccessibility of minerals (macro- and microelements) in tomato farmers’ varieties. They found that among the macroelements, K was the most abundant mineral, followed by Mg, Ca, and Na. Regarding the microelements, the most abundant were Fe and Cu in yellow tomato and Zn and Mn in the round tomato had. The in vitro bioaccessibility assessment showed that, among the macroelements, Mg was more bioaccessible than Ca and K when all the tomato varieties were considered together. Among the microelements, Cu seemed to be the most bioaccessible. Although the contribution of 100 g of tomato shows a relatively low content of minerals according to the dietary reference intakes, tomatoes could contribute to reaching these mineral requirements, as it is included in the diet of most of the population, especially in Mediterranean regions.

On another hand, Szpunar-Krok et al. [5] evaluated nutritional composition and health-promoting fat in seeds of soybean under the influence of climatic conditions, varying doses of N and inoculation with *Bradyrhizobium japonicum*. The authors found that the level of N fertilizer application and pre-sowing inoculation of seeds of *Bradyrhizobium japonicum* have no effect on the indices. However, climatic conditions were the determinants of all the indicators of soybean oil quality assessment. The course of plant vegetation growth in very warm and relatively humid conditions allowed seeds with the highest content of Omega 3 acids and Omega 6/Omega 3 ratio to be obtained. Thus, results are relevant when considering that soybean oil is the second vegetable oil consumed worldwide.

To close that group of articles, Grubišić et al. [6] explored the effect of wheatgrass juice addition to other fruit juices (apple, beet, carrot, orange, and lemon) on total and in vitro bioaccessibility of minerals, phenolic and flavonoid, and antioxidant activity. The authors found that, after the addition of wheatgrass juice, Ca, Mg, Mn, and Zn concentration increased in all examined juices, vitamin C concentration increased in apple, beet, and carrot juice, total phenolic content increased in carrot juice, while total flavonoid content increased in apple, carrot, and orange juice. The research concludes that, in comparison to the examined juices, wheatgrass juice has better nutritional value, and it could be used in a mixture with other juices to improve their nutritional value.

The remaining portion of articles published under the scope of this Special Issue are reviews that summarize the current evidence of the beneficial effect on health of yoghurts and probiotic fermented milks [7] and organosulfur compounds from onion (*Allium cepa*) [8]. Hadjimbei et al. [7] summary the current evidence of the beneficial effects of yoghurts and probiotic fermented milks. The authors conclude that there exists abundant evidence that the consumption of yoghurt and probiotic fermented milks have a positive health effect in several pathological conditions such as osteoporosis, cardiovascular diseases, and diabetes, alongside the promotion of gut health and the modulation of the immune system. Moreover, the authors propose yoghurts and yogurt products as excellent vehicles for delivering functional ingredients because they are widely accepted and consumed. 

Finally, Guillamón et al. [8] performed the first systematic review about the benefits on the gut microbiota and intestinal health by *Allium cepa* products, specifically by organosulfur compounds from onion. The systematic review shows that organosulfur compounds from onion have shown a significant antibacterial activity against a broad spectrum of antibiotic-resistant bacteria. In addition, some evidence shows that the intake of organosulfur compounds from onion was able to modulate the composition of gut microbiota, increasing the beneficial bacterial populations in animal models. In addition, some evidence suggest that these compounds could be suitable candidates for the treatment of inflammatory bowel disease or reverse the dysbiosis caused by a high-fat diet.

In summary, those seven papers published in this Special Issue are a significant contribution and helped to deeper understanding of the topic of “Nutrient-rich foods for a healthy diet. Volume-I”. These documents are an example of the efforts of researchers in this field and emphasize the need for further research. In fact, the Special Issue “Nutrient-rich foods for a healthy diet. Volume-II” is currently available. We encourage authors to share their valuable contributions in this second Special Issue. 

## Data Availability

The data that support the findings of this study are the articles published in the aforementioned Special Issue. These data were derived from the following resources available in: https://www.mdpi.com/journal/foods/special_issues/Nutrient_Rich_Foods_Healthy_Diet (accessed on 18 July 2023).
